# Abundant L-type calcium channel Ca_v_1.3 (α1D) subunit mRNA is detected in rod photoreceptors of the mouse retina via in situ hybridization

**Published:** 2007-05-23

**Authors:** Hailian Xiao, Xiaoming Chen, Ernest C. Steele

**Affiliations:** Neuroscience Institute, Department of Anatomy and Neurobiology, Morehouse School of Medicine, Atlanta, GA

## Abstract

**Purpose:**

Mutations in the *CACNA1F* gene encoding the L-type calcium channel pore-forming Ca_v_1.4 (α1F) subunit in humans result in an incomplete form of congenital stationary night blindness (CSNB2) with residual photoreceptor function. It has been postulated that this residual function, at least in part, may be mediated by another L-type calcium channel subunit, Ca_v_1.3 (α1D), expressed within cone photoreceptors. However, the expression of the calcium channel Ca_v_1.3 (α1D) subunit within photoreceptors remains debatable due to discrepancies among the immunohistochemical studies reported in the literature. In order to get around the innate complications of utilizing unproven antibodies and to shed light on this discussion, we investigated the mRNA expression profile for the Ca_v_1.3 (α1D) subunit in the mouse retina.

**Methods:**

In situ hybridization was performed on wild type mouse retinal sections with two independent sets of digoxigenin-11-UTP-labeled Ca_v_1.3 (α1D)-specific sense and antisense cRNA probes. The two probe sets employed correspond to two distinct regions of the Ca_v_1.3 (α1D) subunit mRNA, each encoding a different fragment of the Ca_v_1.3 (α1D) polypeptide. In situ hybridization of wild type mouse brain sections with these same probes was performed as an additional control for specificity.

**Results:**

Abundant L-type calcium channel Ca_v_1.3 (α1D) subunit mRNA expression was confirmed in most cells of the outer nuclear layer using two independent Ca_v_1.3 (α1D)-specific antisense cRNA probes, confirming expression in rod photoreceptors. Ca_v_1.3 (α1D) mRNA expression was also observed within most cells of the inner nuclear layer and ganglion cell layers using these same antisense cRNA probes. No labeling of tissue was observed using either sense cRNA probe. In situ detection of concentrated Ca_v_1.3 (α1D) mRNA expression within the hippocampus and Purkinje and granule cells of the cerebellum of wild type mouse brain with these same probes confirmed specificity of the probes.

**Conclusions:**

Our finding of expression of the L-type calcium channel Ca_v_1.3 (α1D) subunit mRNA in rods substantiates the possibility that this pore-forming subunit may be a competent component of channels mediating the residual photoreceptor responses observed in mutant mice lacking functional Ca_v_1.4 (α1F) subunits and in humans with CSNB2. Furthermore, the combined observations of abundant expression of Ca_v_1.3 (α1D) mRNA in wild type rods and the large reduction in the transmission of photoreceptor responses in mice lacking Ca_v_1.4 (α1F) raises the possibility that Ca_v_1.3 (α1D) protein expression levels, localization, or functioning might be concomitantly altered by disruption of the Ca_v_1.4 (α1F) subunit in rods. To date, no studies of Ca_v_1.3 (α1D) mRNA nor protein expression levels or localization in *cacna1f* mutant mice or humans with CSNB2 have been published. Our findings warrant such studies to address the abovementioned possibilities. Finally, the observation of Ca_v_1.3 (α1D) mRNA expression in multiple retinal cell types suggests the potential for a broader role for this L-type calcium channel subunit in overall functioning of the normal retina than previously appreciated. We therefore suggest that lesions in either the gene encoding the L-type calcium channel Ca_v_1.3 (α1D) subunit or other molecules that interact with and regulate it may underlie one or more retinopathies with currently unidentified molecular etiologies.

## Introduction

Incomplete X-linked congenital stationary night blindness (CSNB2) is a recessive, nonprogressive visual disease in humans. CSNB2 was originally described as a distinct form of night blindness characterized by an extremely reduced (negative type) electroretinogram (ERG) b wave due to impaired transduction of the photoreceptor response to second order neurons of the retina [1]. Mutations in the human CACNA1F gene encoding the pore-forming Ca_v_1.4 (α1F) subunit of voltage gated L-type calcium channels have been firmly linked to CSNB2 and are thought to be the underlying molecular lesion causing CSNB2 [2,3]. The immunohistochemical localization of this subunit to putative active zones of the terminals of rod photoreceptors [4,5] of the rat retina supports this notion. The recently reported histological and ERG abnormalities observed in transgenic mice in which the *cacna1f* gene is disrupted [6] and in the naturally occurring *cacna1f* mutant mouse, *nob2* [7], add further support for this hypothesis.

In a recent communication, Morgans et al. [8] suggested that the residual photoreceptor function observed in *nob2* mice and humans with CSNB2 may be the result of rod signals transferred to cones through rod-cone coupling and subsequent forwarding via neurotransmitter release from the cone synapse controlled by another L-type calcium channel subunit isoform, such as Ca_v_1.3 (α1D). In this same report, Morgans et al. demonstrate Ca_v_1.3 (α1D) immunoreactivity in wild type mouse cone pedicles, consistent with previously reported immunohistochemical localization of Ca_v_1.3 (α1D) to cone terminals within tree shrew retina [9,10]. In this same communication [8], however, Morgans et al. acknowledge a second and more parsimonious explanation: the presence of another calcium channel α1 subunit isomer within rod photoreceptors that may mediate neurotransmitter release from the terminals of rods.

Although Morgans' immunohistochemical data support the expression of Ca_v_1.3 (α1D) in mouse cones and not rods, Xu et al., using a different anti-Ca_v_1.3 (α1D) antibody, reported intense immunohistochemical labeling of rod somata in the rat retina [11]. There are 2 potential explanations for the discrepancy in these reports regarding the localization of Ca_v_1.3 (α1D) within photoreceptors. As Morgans et al. [8] examined mouse retina and Xu et al. [11] examined rat retina, the difference may simply reflect a species variation in calcium channel subunit expression. This difference in results could also be the consequence of using different antibodies. As the antibodies used in the two studies were generated against distinct regions of the Ca_v_1.3 (α1D) subunit, they may actually detect different Ca_v_1.3 (α1D) polypeptides translated from different mRNA spice variants of the Ca_v_1.3 (α1D) gene. Such reliance upon antibodies, the specificity of which has not been rigorously demonstrated using tissues from mice with a targeted deletion of the *cacna1d* gene encoding the Ca_v_1.3 (α1D) subunit, is a limitation of both studies. For this reason, we decided to investigate the mRNA expression profile of Ca_v_1.3 (α1D) within the mouse retina using in situ hybridization, with the goal of enlightening the discussion of its potential contribution to forwarding of light signals from rod photoreceptors to second order neurons within the retina. Portions of this study have been previously reported in abstract form (Steele, EC Jr. Analysis of L-type calcium channel Ca_v_1.3 subunit expression in the mouse retina. Invest Ophthalmol Vis Sci 2006; 47:E-Abstract 1046).

## Methods

### Animals

Wild type C57Bl/6J mice were obtained from Jackson Lab for these studies. All animal procedures conformed to the humane treatment of animals as prescribed by the Institute for Laboratory Animal Research (Guide for the Care and Use of Laboratory Animals) and by the Association for Research in Vision and Ophthalmology (ARVO) and were approved by the Morehouse School of Medicine Institutional Animal Care and Use Committee.

### In situ hybridization of retinal and brain sections

Eyes and brains were immediately dissected from animals following euthanasia. Corneas and lenses were immediately dissected from freshly enucleated eyes. Eyecups were then fixed by immersion in 1X phosphate buffered saline (PBS) containing 4% paraformaldehde and diethylpyrocarbonate (DEPC) for 15 min at room temperature. Brains were transected along the midline and fixed by immersion in the same fixative overnight at 4 °C. Eyecups and brains were then rinsed 3 times for 5 min each at room temperature with 1X PBS containing DEPC before they were cryoprotected in 1X PBS containing 30% sucrose and DEPC at 4 °C overnight.

Cryprotected eyes and brains were mounted in Neg50 (Richard Allen Scientific) and flash frozen in liquid nitrogen. A Microm HM 550 OMVP cryostat was then used to cut 14 μm sections, which were subsequently mounted onto Superfrost Plus coated slides (Fisher Scientific).

Total RNA was isolated from 2 wild type C57BL/6J retinae using Trizol Reagent (Invitrogen) according to the manufacturer's specifications and mixed. Two independent fragments of the L-type calcium channel Ca_v_1.3 (α1D) subunit RNA were amplified from total murine retinal RNA using Ca_v_1.3 (α1D)-specific oligos in conjunction with the One-Step RT-PCR amplification kit (Invitrogen). Probe A corresponded to nucleotides 189-715 of GenBank accession number NM_028981 and was amplified using the following oligos: sense, 5'-AGG CAA ACT ATG CAA GAG GCA CCA G-3' and antisense, 5'-ATT CCA TCC ATT CCT AAC GTA AGC-3'.

Probe B corresponded to nucleotides 2486-2742 of GenBank accession number NM_028981 and was amplified using the following oligos: sense, 5'-AAC AAA CCA GAA GTC AAC CAG ATA GCC-3' and antisense, 5'-GGG TTG GTC TTG CTA AGA ATG AAG-3'. These fragments were subcloned into the pCRII-TOPO vector using the TOPO TA Cloning Kit Dual Promoter (Invitrogen) and sequenced to confirm identity. These constructs were then linearized and used to generate antisense and sense probes with the SP6/T7 DIG RNA Labeling kit (Roche) and digoxigenin-11-UTP (Roche) according to the manufacturer's specifications. Probe labeling efficiency was assayed according to the manufacturer's specifications by preparing serial dilutions and spotting onto a nitrocellulose membrane and detecting with alkaline phosphatase-conjugated anti-DIG antibody. The resulting intensities were then used according to the labeling kit manufacturer's specifications to estimate labeled probe concentrations.

Slides with freshly cut sections were air dried for 30 min at room temperature and then prehybridized for 2 h at room temperature in a humidified chamber with hybridization buffer composed as follows: 50% formamide (Sigma-Aldrich), 5X SSC, 5X Denhardt's solution (Invitrogen), 250 μg/ml MRE 600 tRNA (Roche), DEPC-treated water. Hybridization buffer containing about 500 ng /ml DIG-11-UTP-labeled RNA probe, which was preheated to 80 °C for 5 min and then placed on ice, was added to sections before covering with DEPC-treated coverslips and incubating overnight at 61 °C (Probe A) or 58 °C (Probe B) in a humidified chamber. For each probe pair, a range of hybridization temperatures 55 °C, 58 °C, and 61 °C was tested to empirically determine the hybridization temperature which yielded strong staining of tissues with antisense probe following a short colorometric detection reaction time but no staining with sense probe following the same colorometric detection reaction time.

Following hybridization, slides were washed in 0.2X SSC 2 times for 30 min 1 h at 68 °C to remove unbound probe. Slides were then treated with RNase (10 mg/ml) in 0.5M NaCl, 10 mM Tris pH7.5, 5 mM EDTA for 30 min at 37 °C. After RNase treatment, slides were rinsed with 0.2X SSC for 5 min at room temperature, followed by a rinse in 0.1 M Tris pH7.5, 0.15 NaCl for 5 min at room temperature. Slides were then blocked in 0.1 M Tris pH7.5, 0.15 NaCl containing 1-10% heat inactivated normal goat serum for 1 h at room temperature. Slides were then incubated in this same blocking buffer containing alkaline phosphatase-conjugated anti-DIG antibody (diluted 1:400) for 2 h at room temperature. Following incubation with antibody, slides were rinsed 3 times for 7 min with blocking buffer at room temperature to remove unbound antibody. Slides were then equilibrated with a buffer containing 0.1 M Tris pH 9.5, 0.1 M NaCl, 50 mM MgCl_2_ for 5 min at room temperature, before staining with BCIP/NBT in the dark (Vector Labs) according to the manufacturer's recommendations. The colorometric development reaction was stopped by rinsing with TE Buffer (10 mM Tris, 1 mM EDTA, pH 8.0), then with water.

Stained slides were mounted with Vectashield mounting medium (Vector Labs) and images were acquired using a Zeiss Axioskop 2 plus overhead microscope equipped with a Zeiss Axiocam HRc CCD camera with AxioVision acquisition and analysis software. Microsoft Powerpoint and Adobe Photoshop were subsequently employed to assemble images into final figure format.

## Results

### In situ hybridization of murine retinal sections confirms expression of L-type calcium channel Ca_v_1.3 (α1D) subunit in rod photoreceptors of the outer nuclear layer as well as the inner nuclear and ganglion cell layers

**Figure 1:**
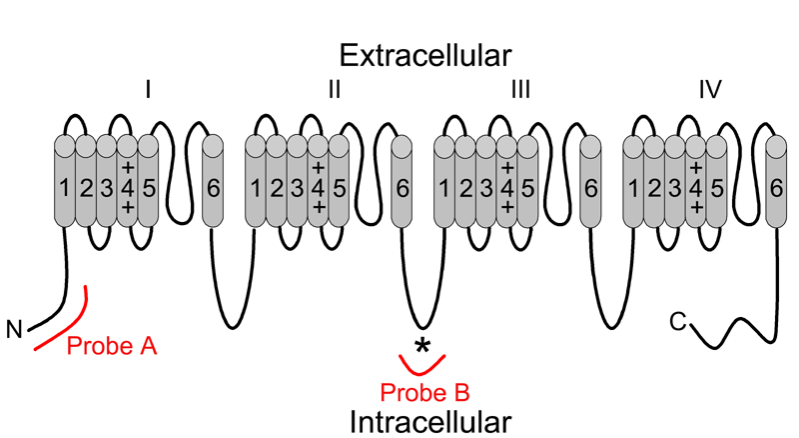
Topological correspondence of Ca_v_1.3 (α1D) in situ hybridization probes. Voltage-dependent calcium channel pore-forming α1 subunits are composed of a single polypeptide consisting of 4 repeating domains (I-IV) joined by intracellular loops. Each repeat domain consists of 6 transmembrane-spanning regions (1-6) connected by intracellular and extracellular loops. Antisense and sense probe sets were generated using cRNA corresponding to 2 different topological regions of the Ca_v_1.3 (α1D) subunit polypeptide. Probe A corresponded to cRNA encoding an N terminal fragment of the polypeptide, while **B** corresponded to cRNA encoding a fragment of the variable loop between repeat domains II and III. Probe B encompassed the antigenic peptide (*) used to generate the commercially available antibody used in most published immunohistochemical studies of Ca_v_1.3 (α1D) expression.

We utilized two independent sets of Ca_v_1.3 (α1D)-specific cRNA probes, corresponding to mRNA encoding 2 distinct regions of the L-type calcium channel Ca_v_1.3 (α1D) subunit, in our in situ hybridization analyses of wild type murine retinal sections. One set of sense and antisense probes, designated as Probe A in Figure 1, corresponds to a 5' fragment of the mRNA encoding an N-terminal fragment of the Ca_v_1.3 (α1D) subunit. The second set of sense and antisense probes, designated as Probe B in Figure 1, corresponds to an internal fragment of the mRNA encoding a fragment of the intracellular loop between the repeat domains II and III of the polypeptide. Probe B encompasses the peptide used as antigen to generate the commercially available anti-Ca_v_1.3 (α1D) antibody employed in many of the previously published immunohistochemical studies of retina, making our mRNA results directly comparable to these studies. Both sets of these probe sequences were blasted against the GenBank databases to confirm their specificity for the Ca_v_1.3 (α1D) subunit and to demonstrate no cross homology with the other calcium channel α1 subunit isomers.

**Figure 2:**
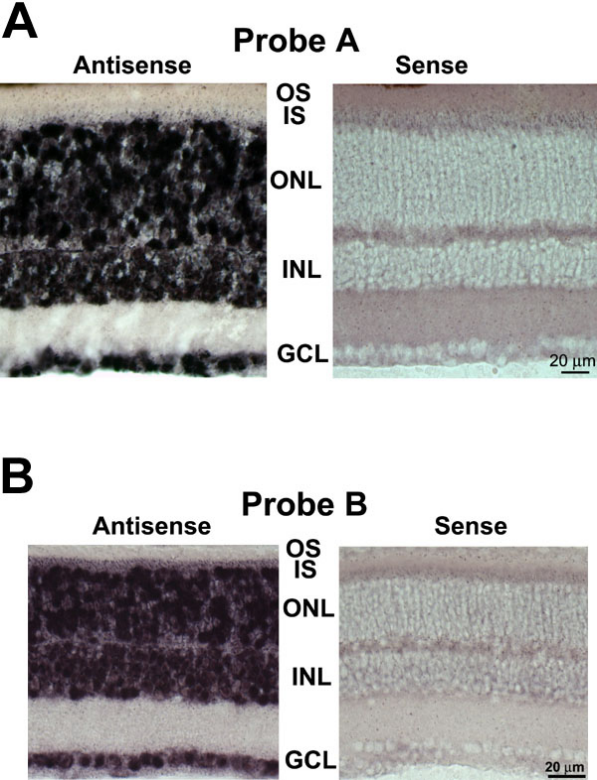
In situ hybridization analysis of Ca_v_1.3 (α1D) mRNA expression in mouse retinal sections confirms expression in most retinal neurons, including rod photoreceptors. **A**: (Probe A) Retinal sections from wild type C57BL/6J mice were hybridized with **A** antisense and sense probes. Antisense probe (left panel) detected Ca_v_1.3 (α1D) mRNA in most, if not all, cells of all three nuclear layers. Sense probe (right panel) did not detect Ca_v_1.3 (α1D) mRNA in any cells of any nuclear layer. IS represents inner segments; OS represents outer segments; ONL represents outer nuclear layer; INL represents inner nuclear layer; GCL represents ganglion cell layer. **B**: (Probe B) In situ hybridization analysis of Ca_v_1.3 (α1D) mRNA expression in mouse retinal sections confirms expression in most retinal neurons, including rod photoreceptors. Retinal sections from wild type C57BL/6J mice were hybridized with **B** antisense and sense probes. Antisense probe (left panel) detected Ca_v_1.3 (α1D) mRNA in most, if not all, cells of all three nuclear layers. Sense probe (right panel) did not detect Ca_v_1.3 (α1D) mRNA in any cells of any nuclear layer.

As shown in Figure 2A and Figure 2B, Ca_v_1.3 (α1D) mRNA expression was detected in the majority of the cells of the outer nuclear layer of mouse retina using antisense cRNA corresponding to the Probe A Figure 2A or the Probe B (Figure 2B) region of the Ca_v_1.3 (α1D) mRNA. Both antisense cRNA probes also detected Ca_v_1.3 (α1D) mRNA within most, if not all, cells of the inner nuclear and ganglion cell layers. We detected no expression of Ca_v_1.3 (α1D) mRNA, however, when sense strand cRNA probes corresponding to these same regions of the Ca_v_1.3 (α1D) mRNA were used. These results were consistent in multiple experiments from multiple animals (Probe A: 4 animals, 9 experiments; Probe B: 6 animals, 10 experiments).

### Specific in situ detection of Ca_v_1.3 (α1D) mRNA in mouse hippocampus and cerebellum confirms specificity of both in situ hybridization probe sets

**Figure 3:**
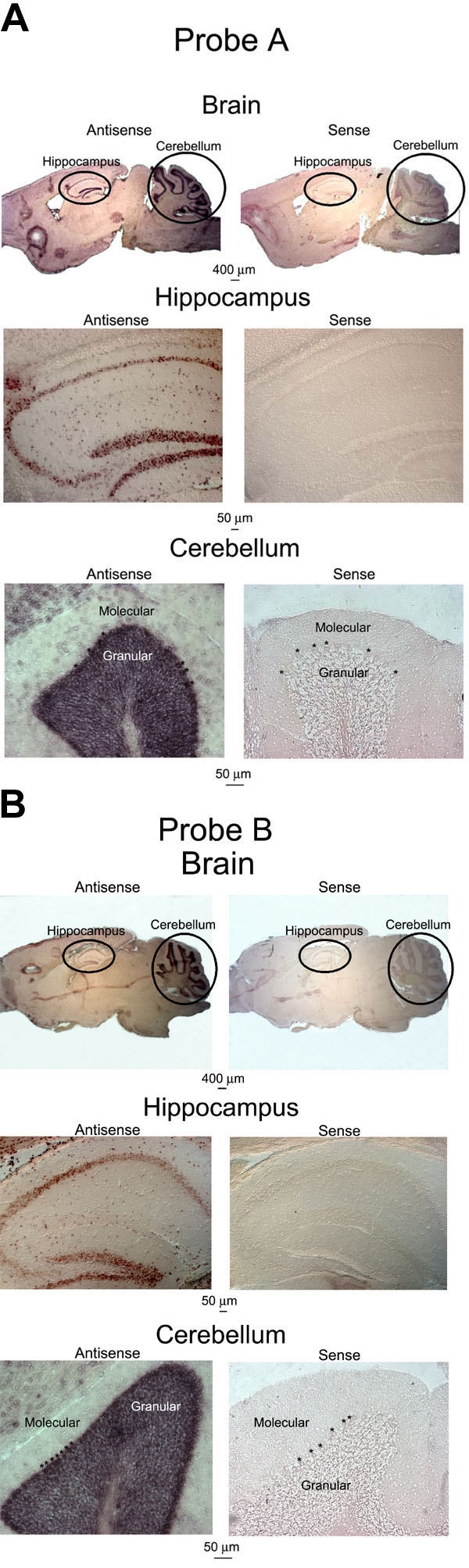
In situ hybridization analysis of mouse brain sections confirms specificity of Ca_v_1.3 (α1D) cRNA probes. **A**: Brain sections from wild type C57BL/6J mice were hybridized with Probe A antisense and sense probes. Antisense probe (left panels) detected Ca_v_1.3 (α1D) mRNA specifically in the hippocampus (middle panel) and cerebellum (bottom panel). In the cerebellum, strong labeling was observed in the large Purkinje neurons (large somas between molecular and granular cell layers, just below asterisks, *, in bottom panels) and in neurons within the granular layer, but was absent from cells of the molecular layer. Sense probes (right panels) did not detect Ca_v_1.3 (α1D) mRNA in any cells of the hippocampus (middle panel) or cerebellum (bottom panel). The observation of such region-specific expression of Ca_v_1.3 (α1D) mRNA in the brain supports the specificity of the Ca_v_1.3 (α1D) probes. **B**: Brain sections from wild type C57BL/6J mice were hybridized with Probe B antisense and sense probes. Antisense probe (left panels) detected Ca_v_1.3 (α1D) mRNA specifically in the hippocampus (middle panel) and cerebellum (bottom panel). In the cerebellum, strong labeling was observed in the large Purkinje neurons (large somas between molecular and granular cell layers, just below asterisks, *, in bottom panels) and in neurons within the granular layer, but was absent from cells of the molecular layer. Sense probes (right panels) did not detect Ca_v_1.3 (α1D) mRNA in any cells of the hippocampus (middle panel) or cerebellum (bottom panel). The observation of such region-specific expression of Ca_v_1.3 (α1D) mRNA in the brain supports the specificity of the Ca_v_1.3 (α1D) probes.

Because most, if not all, cells within each lamina of the retina appear to be expressing some Ca_v_1.3 (α1D) mRNA, the specificity of the probes was called into question. In order to address this, we performed in situ hybridization on sections of wild type mouse brain, where the expression of Ca_v_1.3 (α1D) has been previously reported to be concentrated in neurons of the hippocampus and cerebellum [12]. As shown in Figure 3A and Figure 3B, we observed a virtually identical concentrated expression of Ca_v_1.3 (α1D) within subsets of neurons within the hippocampus and cerebellum using antisense cRNA corresponding to the Probe A (Figure 3A) or Probe B (Figure 3B) region of Ca_v_1.3 (α1D) mRNA. We detected no expression of Ca_v_1.3 (α1D) mRNA in these regions, however, when sense strand cRNA probes corresponding to these same regions of the Ca_v_1.3 (α1D) mRNA were used (Figure 3A,B). These results were obtained in two independent experiments using both Probe sets A and B on brain tissue from two unrelated mice. The detection of Ca_v_1.3 (α1D) mRNA within specific groups of neurons of the brain is in contrast to the more ubiquitous expression profile in the retina and supports the specificity of the Ca_v_1.3 (α1D) probes used in this study.

## Discussion

A limitation of the previously reported immunohistochemical studies of the localization of Ca_v_1.3 (α1D) is the reliance upon anti-Ca_v_1.3 (α1D) polyclonal antibodies based upon the original one of Hell et al. [13]. The antigenic sequence utilized to generate these antibodies is a 20 amino acid sequence corresponding to a unique peptide located within the intracellular loop between repeat domains II and II of the Ca_v_1.3 (α1D) polypeptide and was derived from the rat brain Ca_v_1.3 (α1D) polypeptide sequence. As with most peptide antigen-based antibodies, the anti-Ca_v_1.3 (α1D) antibodies may have limitations to their usefulness, particularly since specificity has not been proven on tissues from the Ca_v_1.3 (α1D) null animals generated by Platzer et al. [14]. In order to avoid these complications, we chose to examine mRNA analysis and utilize two probe sets corresponding to two different regions of the mRNA sequence encoding two distinct regions of the polypeptide.

While Xu et al. previously reported immunolocalization of Ca_v_1.3 (α1D) protein to rod photoreceptor somata in rat retinal sections, it could not be ruled out that this staining represented processes of the Müller cells ensheathing the rod somata [11]. Using an antibody generated against a different region of the Ca_v_1.3 (α1D) subunit, Morgans et al. detected immunoreactivity in cone pedicles [8]. The prominent labeling of most, if not all, cells in the outer nuclear layer of retinal sections in the present study confirms the expression of the Ca_v_1.3 (α1D) calcium channel subunit in rod photoreceptors. The very small numbers of cones in the mouse retina preclude us from making any comment regarding the expression of Ca_v_1.3 (α1D) mRNA in cones or the potential contribution of Ca_v_1.3 (α1D) within cones to transmission of light signals derived via rod-cone coupling to second order neurons of the retina.

The observation of Ca_v_1.3 (α1D) mRNA expression in rods raises some other interesting possibilities worthy of serious consideration in interpreting the reported observations from *cacna1f* null mice [6] and the *nob2* mutant mice with a naturally occurring mutation in *cacna1f*, the gene encoding Ca_v_1.4 (α1F) [7]. Specifically, it warrants concern that the Ca_v_1.3 (α1D) subunit protein expression level, localization, or functioning might be altered in addition to that of Ca_v_1.4 (α1F). Ca_v_1.3 (α1D) protein expression, localization, or functioning may be coupled to Ca_v_1.4 (α1F) expression, localization, or functioning, and be compromised as a direct result of the Ca_v_1.4 (α1F) mutations and concomitant aberration in rod-bipolar cell synapse formation.

Along this same vein of thinking, it is interesting that mice null for the auxiliary b_2_ subunit of voltage-dependent calcium channels have a disrupted outer plexiform synaptic layer and have abnormal ERG b waves. Consistent with the established role of auxiliary βsubunits in targeting the pore-forming a subunits to the plasma membrane [15], the phenotype of the β2 null mice is presumed to be due to the observed inefficiency in targeting of Ca_v_1.4 (α1F) subunits to the synapses of rod photoreceptors. In contrast to mice null for the β2 subunit, mice null for the β1, β3, or β4 subunit do not exhibit histopathological or functional consequences in the retina [16], suggesting that the β2 auxiliary subunit plays the most prominent role in targeting the pore-forming calcium channel α1 subunits to the membranes of the synapses within the retina. To date, no data regarding the identity of the auxiliary βsubunit associated with the Ca_v_1.3 (α1D) subunit in photoreceptors has been published. Based upon our finding s in the present study and the evidence from mice null for the calcium channel β2 subunit, it is highly likely that the auxiliary β2 subunit associates with Ca_v_1.3 (α1D) in addition to Ca_v_1.4 (α1F) in rods and that calcium channels containing the Ca_v_1.3 (α1D) subunit are substantially reduced in the retinas of mice lacking the β2 subunit. To date, no studies of Ca_v_1.3 (α1D) mRNA nor protein expression levels or localization in *cacna1f* mutant mice, the β2 null mice, nor *post mortem* retinae from humans with CSNB2 have been published. Our findings warrant such studies to make sure these possibilities are not overlooked.

Finally, in addition to abundant expression in rod photoreceptors, we noted extensive Ca_v_1.3 (α1D) mRNA expression in most, if not all, neurons within all three nuclear layers of the mouse retina. This observation is consistent with the previously published results of Kamphuis et al. [17] using radiolabeled cRNA probes for in situ analysis of rat retinal sections. The observation of extensive labeling of cells within the inner nuclear layer is also consistent with previous reports of immunohistochemical localization of Ca_v_1.3 (α1D) to the terminals of rat bipolar cells [18] and putative Müller cell processes in rat retina [11] and salamander retina [19,20] and single-cell RT-PCR detection of Ca_v_1.3 (α1D) mRNA expression in acutely isolated human Müller cells [21] and mouse AII amacrine cells [22]. Further experiments using combined in situ hybridization and immunocytochemical analysis of retina or acutely dissociated cells with cell specific markers are required for precise demonstration of the expression of Ca_v_1.3 (α1D) mRNA within specific cell types of the inner nuclear layer of the retina. Although not uninteresting, the question of Ca_v_1.3 (α1D) expression in these other cell types is beyond the scope of this study, which was to investigate the potential expression of Ca_v_1.3 (α1D) in rod photoreceptors as a potential explanation for the observed residual photoreceptor function in patients with CSNB2. The apparently ubiquitous expression pattern of Ca_v_1.3 (α1D) mRNA we observed in the mouse retina suggests the potential for a much broader role for this L-type calcium channel subunit in overall functioning of the normal retina than previously appreciated. We therefore suggest that lesions in the gene encoding the L-type calcium channel Ca_v_1.3 (α1D) subunit or other molecules that interact with or regulate the L-type calcium channel Ca_v_1.3 (α1D) subunit may underlie one or more retinopathies with currently unidentified molecular etiologies.
